# Candidate polyanion microbicides inhibit HIV-1 infection and dissemination pathways in human cervical explants

**DOI:** 10.1186/1742-4690-3-46

**Published:** 2006-08-01

**Authors:** Patricia S Fletcher, Gregory S Wallace, Pedro MM Mesquita, Robin J Shattock

**Affiliations:** 1 Centre for Infection, Department of Cellular and Molecular Medicine, St George's, University of London, London, UK

## Abstract

**Background:**

Heterosexual intercourse remains the major route of HIV-1 transmission worldwide, with almost 5 million new infections occurring each year. Women increasingly bear a disproportionate burden of the pandemic, thus there is an urgent need to develop new strategies to reduce HIV-1 transmission that could be controlled by women themselves. The potential of topical microbicides to reduce HIV transmission across mucosal surfaces has been clearly identified, and some agents are currently under evaluation in clinical trials. Many of these "first generation" microbicides consist of polyanionic compounds designed to interfere with viral attachment. Here we have evaluated two candidate polyanion compounds in clinical trials, PRO 2000 and dextrin sulphate (DxS) to determine their safety and efficacy against *in vitro *HIV-1 and HSV-2 infection using cellular and tissue explant models.

**Results:**

PRO 2000 and DxS potently inhibited infection by HIV-1 X4 and R5 isolates when present during viral exposure. However PRO 2000 required 10-fold and DxS 2000-fold more compound to block infection with R5 virus than X4. While both compounds were virucidal for X4 HIV-1, neither was virucidal for R5 virus. PRO 2000 efficiently inhibited infection of cervical explants and dissemination of virus by migratory DC. DxS was less active, able to completely inhibit cervical explant infection, but providing only partial reduction of virus dissemination by DC. PRO 2000, but not DxS, also inhibited HIV-1 binding to DC-SIGN^+ ^cells and *trans *infection of co-cultured target cells. The inflammatory potential of both compounds was screened by measurement of cytokine production from cervical explants, and statistically significant increases were only observed for IL-1β and RANTES following treatment with PRO 2000. Both compounds also demonstrated potent activity against HSV-2 infection of cervical epithelial cells.

**Conclusion:**

Our results demonstrate that PRO 2000 is a potent inhibitor of R5 HIV-1 infection and dissemination pathways in human cervical explants. DxS, while demonstrating significant inhibition of R5 infection, was less active against DC mediated dissemination pathways. PRO 2000 has now entered human phase III efficacy trials.

## Background

The continuing HIV/AIDS epidemic highlights the need for additional effective methods of prevention. Such methods include the development of topically applied microbicides designed to prevent vaginal HIV-1 transmission. Large-scale efficacy trials for five products, involving tens of thousands of women and tens of millions of dollars, are either planned or are already underway [1]. Three of these products (PRO2000, Carraguard, and Cellulose sulphate) are anionic polymers and inhibit HIV-1 infection by preventing virus-cell fusion/attachment [1-3], predominantly through charge-based interactions with the V3 loop of gp120 [4-6]. Despite working through similar mechanisms, entry of these products into efficacy trials has proceeded without side-by-side preclinical assessment to determine their relative efficacy and safety. In addition, Viva Gel (SPL7013, a sulphated dendrimer), thought to work through similar mechanisms, has been entered in early phase I safety trials [7]. The fourth product in phase III trials is a buffering gel (BufferGel) containing polyanionic carbopol, whilst the fifth is based on the novel surfactant C31G (termed SAVVY) [8].

Here we describe the side-by-side preclinical evaluation of two anionic candidates, PRO 2000 and dextrin sulphate (DxS), prior to selection for phase III efficacy trials by the Microbicide Development Programme (MDP-UK). PRO 2000 is a synthetic naphthalene sulphonate polymer (average molecular weight approximately 5 kDa). Early observations suggested binding to CD4 and the V3 region of gp120, blocking subsequent interaction between CD4 and gp120 [9,10] and preventing infection of T lymphocytes, macrophages and cervical explant tissue [9-12]. More recent investigations using surface plasmon resonance (SPR) have suggested gp120 binding may be less dependent upon V3 charge, however they confirm that PRO2000 prevents viral entry [13]. Additional studies have suggested that high concentrations of a polynaphthalene sulphonate (5 mg/ml) can induce gp41 six helix bundle formation [6] rendering the virus non-infectious. DxS is a synthetic sulphated polysaccharide (average molecular weight approximately 20 kDa), whose anti-viral activity is distinct from related dextran sulphate [14-16]. Early studies suggested that DxS binds strongly to tat, and weakly to gp160/120 [17,18]. However, more recent structure function-studies have demonstrated that the predominant activity of DxS is mediated through binding to gp120, regulated by the degree of polymer sulphation and V3 loop charge [15]. Thus, like PRO2000, DxS targets viral entry and both have been shown to inhibit a diverse panel of HIV isolates *in vitro *[16-18]. Furthermore, PRO 2000 and DxS have shown varying levels of protection against a SHIV-89.6 vaginal challenge in the rhesus macaque model [19,20].

We have evaluated both candidates to determine their potential selectivity against R5 and X4 HIV-1 using *in vitro *cell based assays. In addition, the activity of these compounds has been tested in a human cervical explant culture model [12,21] to determine efficacy against both localized infection and dissemination of virus by migratory cells.

## Results

### Differential activity of polyanion microbicides towards X4 and R5 HIV-1

**Figure 1:**
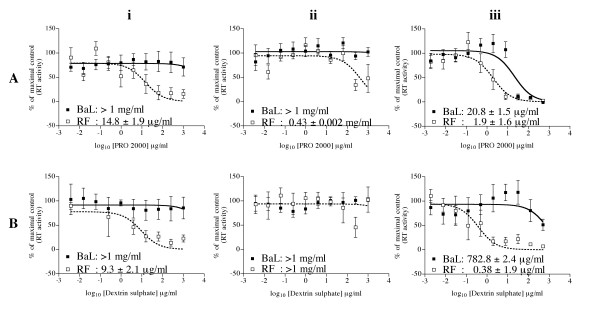
**Inhibitory effect of polyanionic compounds against HIV-1 infection of T-cells**. HIV-1 BaL (R5, ■, solid line) or RF (X4, □, dotted line) was immobilised onto solid phase using anti-HLA-DR antibody capture, as described in the Methods. (i) Direct virucidal activity was determined by the pre-treatment of immobilised virus for 1 hour before culture with target PM-1 cells in the absence of compound. (ii) Receptor mediated blockade activity was determined by the pre-treatment of target PM-1 cells (1 hour) prior to exposure to immobilised virus in the absence of compound. (iii) Attachment/fusion inhibition was determined by the pre-treatment of immobilised virus with test compound prior to the addition of target PM-1 cells in the presence of compound. Plates were cultured for 10 days following which viral replication was determined by reverse transcriptase measurement of culture supernatants. Compounds tested were: A) PRO 2000; and B) Dextrin sulphate. Data represent the mean ± SEM of n = 5 (PRO 2000) or 4 (Dextrin sulphate) independent experiments where each condition was tested in triplicate. Inserted figures represent the mean ± SEM concentration inhibiting 50% infection (IC_50_) for compounds against each virus.

Direct virucidal activity was assessed by compound treatment of immobilised virus, prior to washing and culture with permissive T cells as previously described [22]. Both compounds demonstrated potent activity against the X4 isolate, with 50% inhibitory concentrations (IC_50_) observed at 14.8 (± 1.9) and 9.3 (± 2.1) μg/ml of PRO 2000 and DxS respectively (Figure 1Ai &1Bi). In contrast, both compounds failed to exert any effect against R5 virus, even at concentrations of 1 mg/ml (Figure 1Ai &1Bi). Receptor mediated blockade was assessed by incubating target cells with compound prior to compound removal and culture with immobilised virus; this was poor or absent for both compounds (Figure 1Aii &1Bii). Inhibition of attachment/fusion was assessed by pre-treatment of virus with test compound for 1 hour prior to culture with permissive cells in the presence of compound. Both compounds exhibited potent activity against R5 and X4 infection, although greater activity was observed against X4 than R5 virus with IC_50 _values of 1.9 (± 1.6) and 20.8 (± 1.5) μg/ml respectively for PRO 2000, and 0.38 (± 1.9) and 782.8 (± 2.4) μg/ml respectively for DxS (Figure 1Aiii &1Biii).

### Toxicity of polyanions towards female genital mucosal tissue cultured *ex vivo*

**Figure 2:**
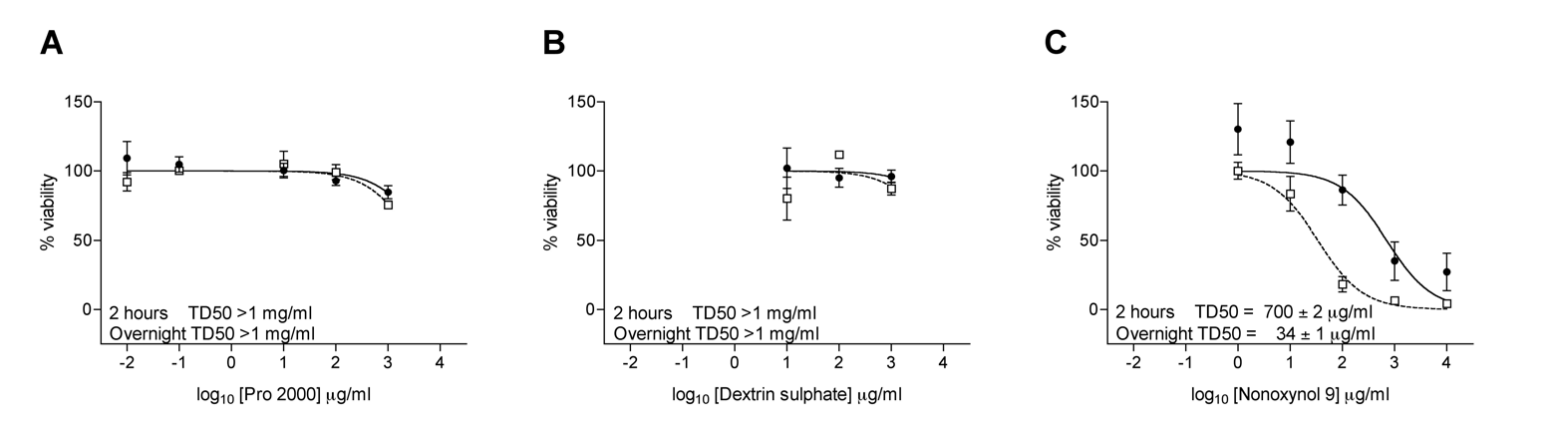
**Toxic effects caused to cervical tissue following compound treatment**. Ectocervical explants were exposed to compounds for 2 or 24 hours. Explant viability was then determined using the principle of MTT dye reduction as described in the Methods section. % viability was calculated per mg tissue comparing compound-treated explants to unexposed controls. Compounds tested were A) PRO 2000; B) Dextrin sulphate; and C) Nonoxynol-9. Data represent the mean ± SEM of n = 4 (PRO 2000), 2 (Dextrin sulphate) or 7 (Nonoxynol-9) independent donors where each condition was tested in triplicate. Inserted figures represent the mean ± SEM 50% toxic dose (TD_50_) following compound treatment for 2 (● solid line) or 24 (□ dotted line) hours.

Before the activity of compounds against HIV-1 infection of female genital tissue was investigated, it was important to ensure that neither compound would elicit a toxic effect. This was evaluated using genital mucosal tissue explants obtained from seronegative women undergoing therapeutic hysterectomy as previously described [12,21]. Tissue explants were immersed in test compound for 2 or 24 hours and tissue viability determined using the principle of MTT dye reduction (see Methods). Compounds were tested to a maximal concentration of 1 mg/ml and toxicity was compared to the known toxic agent Nonoxynol-9 (N9) [23]. Only mild toxic effects were observed with both PRO 2000 and DxS following 2 hour compound treatment, with 50% toxic doses (TD_50_) of greater than 1 mg/ml for both compounds (Figure 2A and 2B). This was in contrast to N9, which caused significant toxicity with a TD_50 _of 700 (± 2) μg/ml following a 2 hour treatment period (Figure 2C). In fact, N9 caused significant toxicity at 1 mg/ml, causing a 65% reduction in viability. Furthermore, 24 hour treatment of tissue with N9 caused significant damage (TD_50 _= 34 ± 1 μg/ml), whilst only mild toxicity was observed following 24 h treatment with either PRO 2000 or DxS (TD_50 _> 1 mg/ml).

### Inhibition of HIV-1 infection of human cervical tissue and dissemination of virus by migratory cells

**Figure 3:**
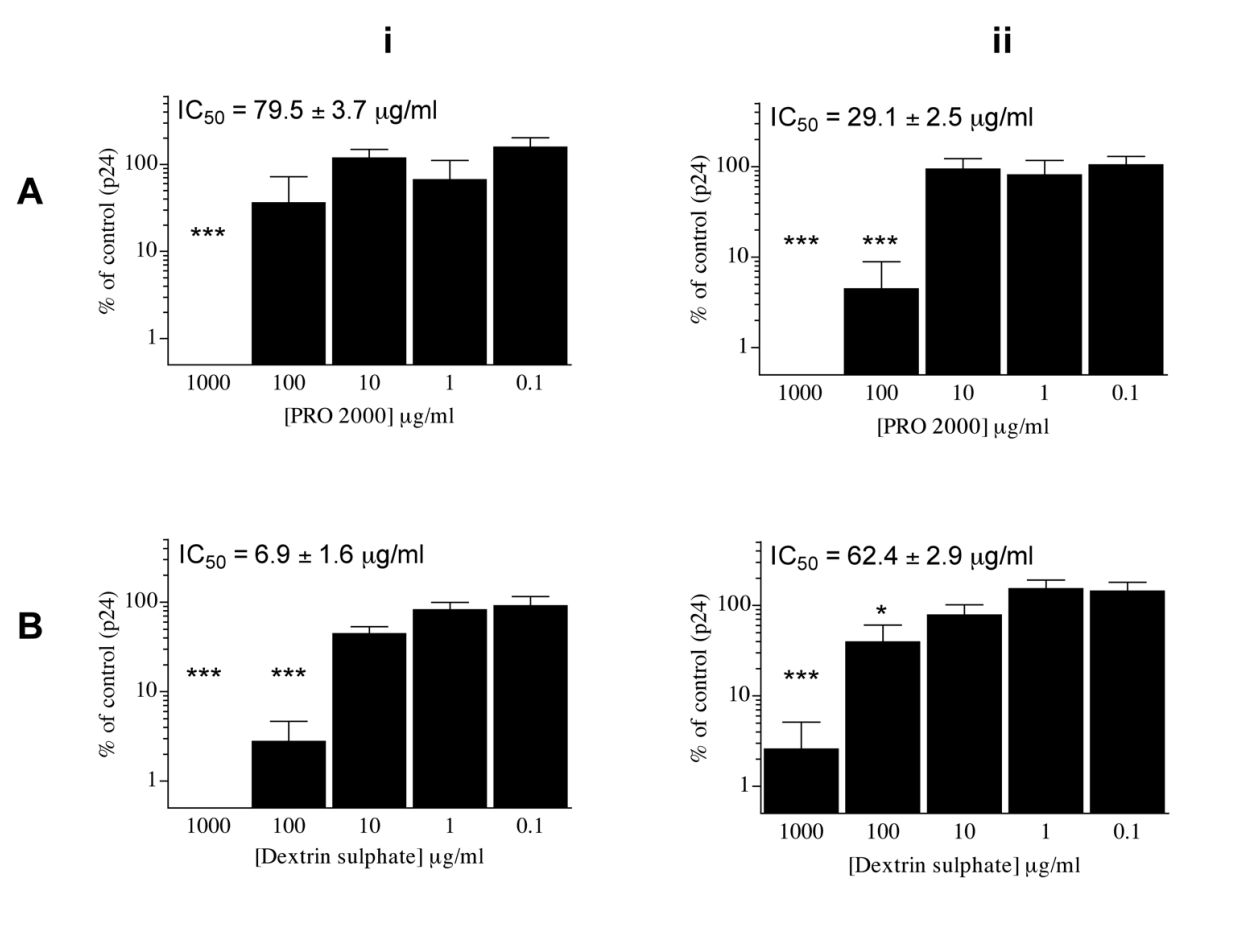
**Polyanion inhibition of HIV-1_BaL _infection of cervical explants and transfer of virus from migratory cells**. Ectocervical explants were exposed to HIV-1_BaL _for 2 hours in the presence of test compound. Following overnight culture, explants were separated from any cells that had migrated from the tissue and cultured separately. (i) Infection of cervical explants was determined by ELISA measurement of p24 antigen in culture supernatants. (ii) Migratory cells were co-cultured with permissive T cells (PM-1) and infection determined by p24 antigen in culture supernatants. Data represent the % HIV-1 infection observed following compound treatment when compared to tissue exposed to virus alone. Each compound was tested using n = 3 – 8 independent donors, where each condition was tested in triplicate. Compounds tested were A) PRO 2000 and B) Dextrin Sulphate. Inserted figures represent the mean ± SEM concentration inhibiting 50% HIV-1 infection (IC_50_) for each compound. Statistical analysis was completed using student's T-test with statistically significant changes marked * (p < 0.05), or *** (p < 0.005).

The potential of PRO 2000 and DxS to block infection of the female genital mucosa was investigated using ectocervical explants, cultured in a non-polarised manner as previously described [21,22]. Explants were treated with test compound (PRO 2000 or DxS) for 1 hour prior to exposure to R5 HIV-1_BaL _for 2 hours in the presence of compound as described in the Methods. Viral infection was evaluated by p24 released into culture supernatants. The activity of polyanions against HIV-1_BaL _infection of cervical explants was dose-dependent (Figure 3). Both PRO 2000 and DxS were able to completely inhibit infection at 1 mg/ml (p < 0.001), but allowed breakthrough of infection to occur at 100 μg/ml, with DxS being 10 fold better than PRO 2000 with an IC_50 _of 6.9 (± 1.6) versus 79.5 (± 3.7) μg/ml (Figure 3i).

We have previously shown spontaneous migration of CD4^+ ^dendritic cells (DC) from cervical explant tissue during overnight culture, a population of cells able to bind virus via mannose C-type lectin receptors (MCLR) and/or CD4 [21]. Migratory cells were harvested from explant cultures (exposed to compound and virus as described) following overnight culture, washed to eliminate cell free virus, and co-cultured with permissive PM-1 T cells. The effect of both compounds in preventing dissemination of virus by these migratory cells was dose-dependent. PRO 2000 completely inhibited viral transfer at 1 mg/ml, and demonstrated significant inhibition (>90%) at 100 μg/ml, with an IC_50 _of 29.1 (± 2.5) μg/ml (Figure 3Aii). DxS provided 95% protection at 1 mg/ml (Figure 3Bii) demonstrating an IC_50 _of 62.4 (± 2.9) μg/ml.

### Inhibition of HIV-1 binding to DC-SIGN and transfer to permissive cells

**Figure 4:**
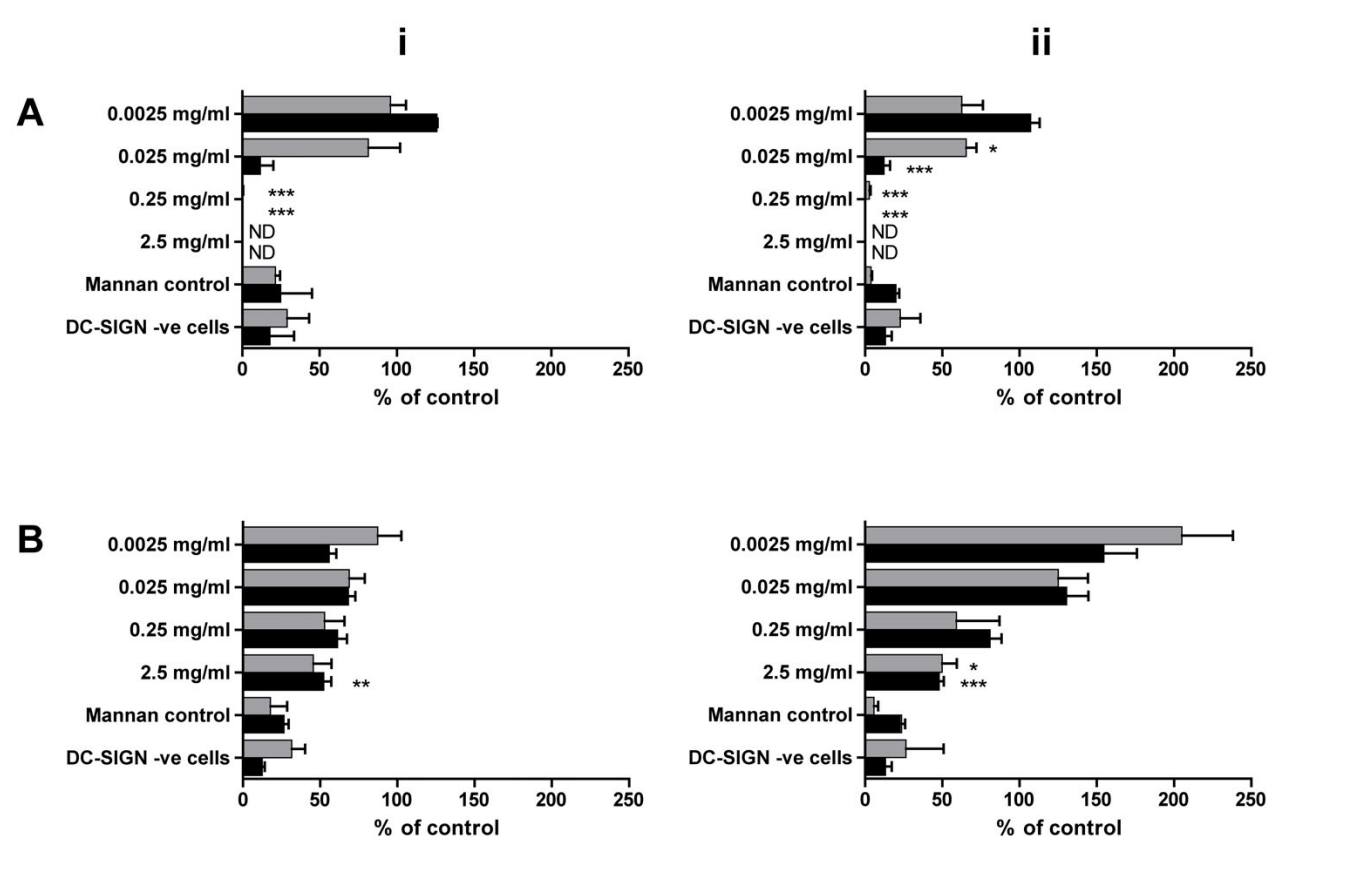
**Inhibition of HIV-1 binding and transfer through DC-SIGN by polyanions**. DC-SIGN^+ ^Raji cells were treated with test compound (A) PRO 2000 or B) Dextrin Sulphate for 1 hour prior to exposure to HIV-1 (i) RF or (ii) BaL for 2 hours in the presence of compound. Following removal of excess compound and virus, cells were either lysed for analysis by p24 ELISA to determine inhibition of viral binding (■), or co-cultured with permissive T cells (PM-1) to determine inhibition of viral transfer (). Background binding to Raji cells was determined using Raji/DC-SIGN^- ^cells, or 100 μg/ml mannan. Data represent the mean ± SEM of 3 independent experiments (except PRO 2000 against RF, where n = 2) where each condition was tested in triplicate. Statistical analysis was completed using student's T-test with statistically significant changes marked * (p < 0.05), ** (p < 0.01) or *** (p < 0.005). ND = not determined.

Having observed that both compounds showed some efficacy against dissemination of HIV-1 by migratory cells, subsequent experiments were carried out to determine whether either compound blocked DC-SIGN binding and/or transfer. To this end, Raji-DC-SIGN^+ ^CD4^- ^cells were incubated with candidate polyanions during exposure to virus (2 h). Excess virus and compound were removed by washing and cells either directly lysed to determine the amount of virus bound to cell surface receptors, or cultured with permissive T cells (PM-1) to assess *trans *infection. Mannan, the natural ligand for DC-SIGN and other MCLR, blocked most, but not all, binding of virus to Raji DC-SIGN^+ ^cells. Viral binding to Raji DC-SIGN^+ ^cells in the presence of mannan (100 μg/ml) mirrored values seen with Raji DC-SIGN^- ^cells (Figure 4), indicating a low level (20% of untreated controls) of DC-SIGN-independent binding of virus to Raji cells. PRO 2000 exhibited significant activity at 0.25 mg/ml against virus binding to DC-SIGN and *trans *infection of PM-1 cells (Figure 4A). DxS exhibited a lower level of inhibition, demonstrating a maximal 50% inhibition of both binding and *trans *infection at the highest concentration of 2.5 mg/ml (Figure 4B) while demonstrating no statistically significant effect at lower concentrations when compared to untreated controls (taken as 100%).

### Effects on pro-inflammatory cytokine response in human cervical tissue

**Figure 5:**
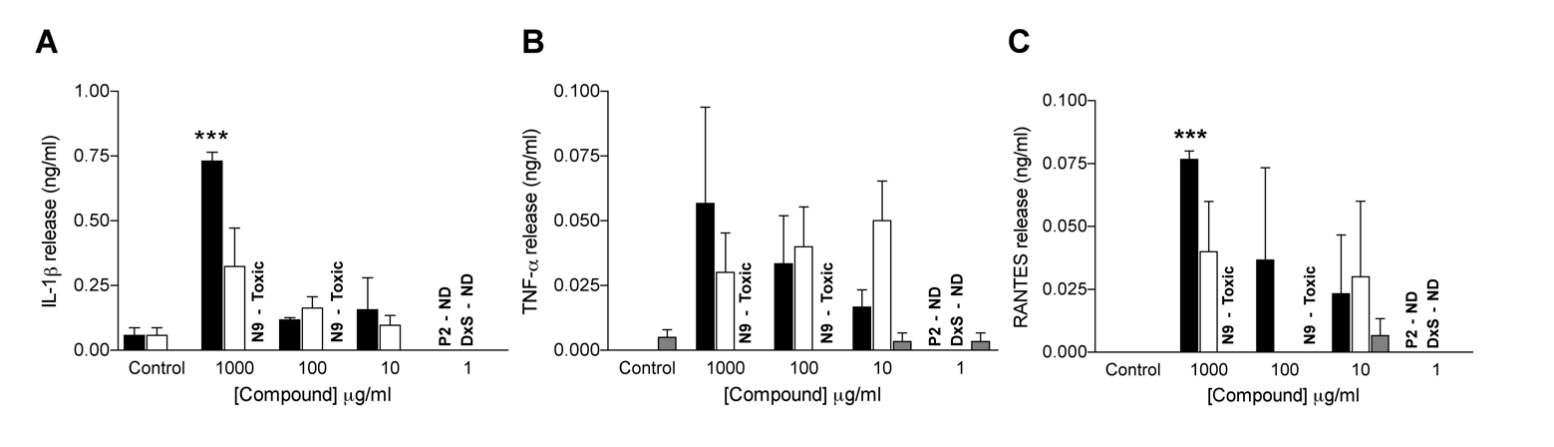
**Stimulation of inflammatory cytokines in cervical tissue treated with polyanions**. Tissue explants were exposed to PRO 2000 (■), Dextrin Sulphate (□) or Nonoxynol-9 () for 2 hours prior to compound removal by washing and overnight culture in the absence of compound. Culture supernatants were assessed (using the Bioluminex assay) for the presence of the cytokines: A) IL-1β; B) TNF-α; and C) RANTES. Data represent the mean ± SEM for 3 individual donors. Statistical analysis was completed using student's T-test with statistically significant changes marked *** (p < 0.005). ND = Not determined. Toxic = Compound treatment caused >50% reduction in tissue viability.

To investigate whether exposure of human cervical tissue to candidate polyanions would elicit an inflammatory response, tissue explants were exposed to compound (2 h) prior to compound removal by washing and overnight culture. Culture supernatant was assessed by Bioluminex assay for the presence of a panel of 9 cytokines (IL-1β, IL-6, IL-8, TNF-α, GM-CSF, MIP-1α, MIP-1β, RANTES, and MCP-1). Untreated tissue explants produced detectable levels of all cytokines except TNF-α and RANTES, which were towards the limits of detection. Treatment with either compound (1 mg/ml) had little or no effect on the production of most of the cytokines including IL-6, IL-8, GM-CSF, and MIP-1α (data not shown). However, treatment with 1 mg/ml of either PRO 2000 or DxS resulted in a 13 or 6 fold (respectively) increase in IL-1β release (Figure 5i), which was statistically significant (p = 0.006) for PRO 2000. Both compounds also induced increases in TNF-α and RANTES production (Figure 5ii and 5iii) although only the increase in RANTES induced by PRO 2000 reached statistical significance (p = 0.002). To aid the interpretation of this data, results were compared with explants treated with an equal dose of the toxic compound N9. Unfortunately, N9 caused significant (≥ 50%) toxicity to tissue at concentrations of ≥ 100 μg/ml. Although approximately 50% viability was still observed at 100 μg/ml, the effect such toxicity had on cytokine release could not be determined with complete confidence, therefore only concentrations causing no toxicity were used for comparison. In general, treatment of tissue with 10 μg/ml N9 caused little change in cytokine release. To determine whether there was any correlation between increasing compound dose and release of cytokines, data was analysed using Spearman rank correlation and significance determined using two-tailed significance testing of paired samples. However, none of the compounds demonstrated any significant correlation between increasing compound dose and modulation of cytokine release, suggesting the observed cytokine release was unlikely to reflect adverse response to compound treatment.

**Figure 6:**
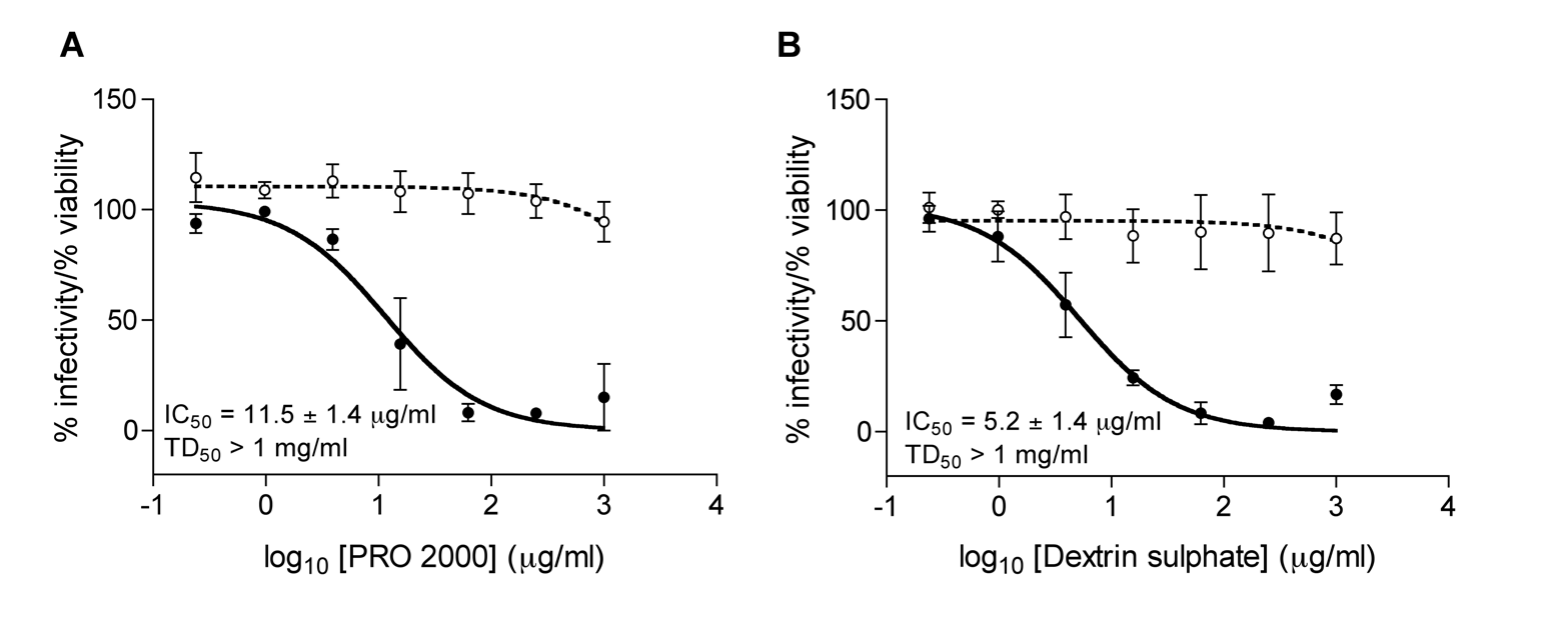
**Inhibitory effect of polyanionic compounds against HSV-2 infection of epithelial cells**. ME180 cells (seeded at 1.5 × 10^4 ^cells/well and cultured overnight), were exposed to HSV-2 (~5 × 10^4 ^Pfu/well) in the presence of compound for 1 hour, or alternatively, exposed to compound alone. Following compound/virus removal by washing, cells were cultured for a further 48 hours when viability was determined by MTT dye reduction. Cell viability (○, dotted line) following compound treatment was calculated as a percentage of the viability of cells exposed to culture medium alone. The effect of compound treatment on the infectivity of HSV-2 (●, solid line) was calculated as a percentage of infection observed in cells exposed to virus alone. Compounds tested were: A) PRO 2000; and B) Dextrin sulphate. Data represent the mean ± SEM of 3 independent experiments where each condition was tested in triplicate. Inserted figures represent the mean ± SEM concentration inhibiting 50% HSV-2 infection (IC_50_) or concentration causing 50% toxicity (TD_50_) towards ME180 cells.

### Inhibition of HSV-2 infection of vaginal epithelial cells

Due to the strong correlation reported between the presence of genital herpes and HIV-1 transmission [24], the effect of both PRO 2000 and DxS on the ability of HSV-2 to infect vaginal epithelial cells was investigated using the ME180 cell line. ME180 cells were exposed to HSV-2 (1 hour) in the presence of test compound and, following compound removal, cells were cultured for 48 hours in the absence of compound and virus, and viability determined by the principle of MTT dye reduction (see Methods). PRO 2000 and DxS demonstrated no significant toxicity towards ME180 cells, and both compounds demonstrated potent anti-HSV-2 activity, with IC_50 _values of 11.5 (± 1.4) μg/ml (PRO 2000) and 5.2 (± 1.4) μg/ml (DxS) (Figure 6).

## Discussion

Successful microbicides will need to prevent all potential mechanisms of mucosal HIV transmission. Whilst blockade of cell surface receptors (CD4, CCR5 and CXCR4) within the mucosa may prevent localised infection of T cells and macrophages, viral uptake and dissemination by DC occurs through CD4 and MCLRs [21]. Thus, preventing HIV-1 infection is highly likely to require compounds able to block viral attachment via multiple cell surface receptors. Furthermore, as HIV-1 transmission has been associated with the presence of other sexually transmitted infections (STIs) [24] such as HSV-2, it may be useful for a topical compound to possess the ability to block such infections. Here we have evaluated the potential of two anionic polymers, PRO 2000 and DxS, to inhibit these different pathways

In agreement with previous studies [9-18], we have demonstrated that PRO 2000 and DxS potently inhibited infection by both X4 and R5 isolates of HIV-1 when present during viral exposure in cell based *in vitro *assays (Figure 1). Interestingly, these products demonstrate similar *in vitro *activity to Viva gel (SPL7013) being fast tracked for clinical trials [7]. However PRO 2000 required 10-fold and DxS 2000-fold more compound to block infection with R5 virus than X4 (Figure 1iii), confirming previous studies demonstrating differential activity against these viral phenotypes [13,17]. In addition, pre-treatment of cells with either compound failed to provide any cellular protection. These observations confirm that activity is not mediated by steric hindrance following binding to CD4 as first thought [9,14], but through binding with gp120, preventing subsequent receptor/co-receptor interaction [4]. While V3 charge may not be the predominant factor regulating binding *per se *of polyanions to gp120 [13], this does not negate previous observations that inhibition itself is mediated by electrostatic interaction with the gp120 V3 loop [4,17]. Competition by polyanions for these sites is more efficient the greater the envelope charge, with X4 isolates being more highly basic (>5+) than R5 isolates (2–5+) [17,25]. This is likely to account for the differential activity of the polyanions seen against X4 and R5 virus in the presence of compound. Furthermore, while both exhibited direct virucidal activity against X4 virus when pre-treated with compound, neither was virucidal for R5 virus at the concentrations tested (1 mg/ml). Such differential activity suggests that X4 isolates could be inactivated by anionic polymers within the vaginal lumen, while R5 virus would require compound to reach target cells within the mucosa with equal efficiency as the virus itself [26]. It is unclear whether the observed virucidal activity against X4 virus was mediated by induction of gp41 six-helix bundle formation. Previous studies demonstrated that 5 mg/ml polynaphthalene sulphonate was required to induce six-helix bundle formation in both X4 and R5 virus [6]. In this study we have evaluated the ability of both compounds against R5 HIV-1BaL as this virus, unlike many primary strains, provides reproducible infection of cervical tissue explants. However, PRO2000 and DxS have shown similar activity against a range of primary stains in different cellular and tissue models [11-13,15,17], suggesting that these results may predict activity against a wider range of virus stains. Interestingly, formulated PRO2000 gel performed similarly to Viva Gel (SPL7013) and better than Carraguard when tested at a single dose against primary strains in a comparable cervical explant model [11].

In contrast, microbicides based on anionic polymers have only been tested against X4 SHIV (SHIV-89.6) in the rhesus macaque vaginal challenge model [19,20]; SHIV 89.6 has sufficient charge to be inactivated by direct electrostatic interaction with polyanions in the vaginal lumen. However, as R5 virus is predominantly associated with HIV transmission [27,28], it will be important to evaluate the efficacy of such compounds against R5 virus (e.g. SHIV-162p) [29], particularly as they will need to cross the mucosa and reach target cells as efficiently as the virus itself. It is unlikely that such high molecular weight compounds can be absorbed across intact cervicovaginal epithelium and this is reflected by lack of detectable systemic toxicity [29,30] and adsorption [31] following vaginal application in human phase I trials. However, an intact stratified epithelium also provides a significant barrier to HIV-1 transmission [12], and infection is most likely associated with epithelial microtrauma [32,33]. It is anticipated that such epithelial damage would also facilitate sufficient penetration of compound to protect localized susceptible cells. To test this hypothesis we have used a non-polarized explant culture system where virus and compound access all potential susceptible cells within the epithelium and underlying mucosa, such as would be the case if a breach to the mucosal surface were to occur.

In the absence of any significant toxicity (Figure 2), both PRO 2000 and DxS inhibited HIV-1 infection of cervical explant tissue, when exposed to virus in the presence of compound, with DxS providing better protection than PRO 2000. We also investigated the effects of both compounds on virus dissemination by DC that spontaneously migrate out of cervical explants. Although both compounds reduced transfer of virus by migratory cells with similar IC_50 _values, only PRO 2000 was able to completely prevent transfer at 1 mg/ml (Figure 3Aii). It was not possible to determine whether *trans *infection of co-cultured T cells was due to uptake of virus by MCLR in the absence of DC infection, or dependent upon prior *cis *infection of DC themselves. Recent studies have suggested that *trans *infection of T cells, independent of DC infection occurs with decreasing efficiency over the first 4–24 hours, while *cis *infection of the DC occurs 24–72 hours following virus exposure [34,35]. Thus in our model it is likely that amplification of virus from migratory DC harvested following overnight culture (approximately 18 hours) occurs through a mixture of both mechanisms.

To determine whether either compound directly affected virus binding to DC-SIGN, parallel experiments were carried out using DC-SIGN^+ ^Raji cells. At 0.25 mg/ml PRO 2000 inhibited both X4 and R5 virus binding to DC-SIGN and also *trans *infection of co-cultured indicator T cells by cell bound virus (Figure 4A). These data suggest that PRO 2000 can block binding to DC-SIGN and/or that sufficient compound remains associated with the cells (or virus) to prevent bound virus being transferred to susceptible T cells. In contrast DxS failed to provide complete inhibition of either virus binding or *trans *infection at the highest dose tested (2.5 mg/ml). These data are in agreement with results obtained from the cervical DC experiments described above and suggest that DxS may be less efficient at preventing HIV dissemination by migratory DC.

Having determined the efficacy of both compounds at non-toxic concentrations in the above models, we then investigated the potential of either compound to elicit pro-inflammatory cytokine production in human cervical tissue. Only increases in IL-1β and RANTES, following exposure to PRO 2000, reached statistical significance. Although Il-1β release has been linked with adverse effects associated with topical application of N9 [36], levels of production reported here showed no significant correlation with increasing compound dose. In fact, inflammatory tissue damage caused by topical application of N9 has been associated with an increase in IL-8 release [36], which was not observed with either PRO 2000 or DxS in this study. Thus these data are unlikely to reflect the occurrence of an adverse response to compound application *in vivo*. Nevertheless, in some (but not all) human phase I clinical trials, mild adverse events were more common with topical application of 4% PRO 2000 than 2% and 0.5% formulations of PRO 2000 [30,37].

In addition to demonstrating anti-HIV activity, it would be advantageous for a microbicide product to demonstrate activity against other STIs. Both PRO 2000 and DxS demonstrated potent activity against HSV-2 infection of cervical epithelial cells with similar efficacy, in agreement with previous reports for PRO 2000 against HSV-2 infection of human endocervical cells [38] or cervical epithelial (CaSki) cells [39]. These data are similar to those reported for Viva Gel (SPL7013) [40], suggesting no competitive advantage for this second generation polyanion. Furthermore, previous reports have suggested that formulated PRO 2000 (0.5% gel) retained *in vitro *anti-viral activity against both HIV-1 and HSV-2 following *in vivo *intravaginal application [39], whilst the 4% gel protected against *in vivo *HSV-2 infection in the cotton rat model [41].

Although formulated concentrations of PRO 2000 and DxS are higher than those required to prevent infection *in vitro*, they are highly likely to be diluted following vaginal application through product leakage prior to intercourse and on mixing with seminal and vaginal secretions. Based on infectivity data derived from the *ex vivo *cervical explant model, formulated PRO 2000 could be diluted 1/200 (2%) or 1/50 (0.5%) before being reduced below its protective range (100 μg/ml). However, for protection against viral dissemination by DC, this would be reduced to 1/20 (2%) or 1/5 (0.5%). In contrast, DxS while preventing cervical explant infection at a dose equivalent to a 1/40 dilution of the 4% formulation, failed to provide complete protection against DC mediated viral dissemination at the highest dose tested.

## Conclusion

In conclusion, these data demonstrate that PRO 2000 and DxS are active against R5 virus in cellular and tissue models. How these *in vitro *results will translate into *in vivo *efficacy is not yet known. The Microbicides Development Programme (UK) has elected to evaluate both 2% and 0.5% PRO 2000 gel in human phase III efficacy trials. In addition, 0.5% PRO 2000 gel will be evaluated by the HIV Prevention Trials Network (Protocol HPTN 035).

## Methods

### Cell culture and reagents

PM-1 (AIDS reagent project, National Institute for Biological Standards and Control, Potters Bar (NIBSC), UK), Raji, Raji/DC-SIGN (provided by V N Kewal-Ramani, HIV Drug Resistance Program, NCI, Frederick, MD) and Vero cells were grown in complete RPMI [RPMI 1640 medium supplemented with 10% fetal calf serum, 100 U/ml penicillin, 100 μg/ml streptomycin and 2 mM L-glutamine]). The adherent cell line ME180 was cultured in DMEM supplemented as complete RPMI (complete DMEM). All cells were grown in continual culture in a humidified environment of 5% CO_2 _at 37°C and passaged every 3–4 days.

HIV-1 strains (HIV-1_BaL _and HIV-1_RF_, AIDS reagent project, NIBSC, UK) were grown in phytohaemagglutinin (PHA)-stimulated peripheral blood mononuclear cells as previously described [12]. Cell-free viral stocks were passed through 0.2 μm pore-size filters. Infection was monitored by viral p24 antigen (HIV-1 p24 ELISA, AIDS Vaccine Program, National Cancer Institute (NCI) at Frederick, MD, USA), carried out according to manufacturers protocol) or reverse transcriptase (RT) [42] release into culture supernatants. The 50% tissue culture infectious dose (TCID_50_) was determined in PM-1 cells for both viruses, and additionally in PHA-stimulated PBMC for HIV-1_BaL_.

HSV-2 (G) (kindly donated by Dr. B. Herold (Mount Sinai School of Medicine, NY, USA)) was grown in Vero cells. Infectivity of viral stocks was assessed by plaque assay using ME180 cells as previously described [43].

Unformulated PRO 2000 was provided by Indevus Pharmaceuticals, USA, and DxS by ML Laboratories, UK. Both products were used at non-toxic concentrations as determined by MTT viability assays.

### Solid-phase immobilisation of HIV-1

Solid phase immobilisation of HIV was carried out as previously described [22]. In brief, HLA-DR Mab (L243, ATCC) was bound to 96 well, flat bottom, tissue culture plates (Nunc) for 1 hour at room temperature. Unbound antibody was washed off with 1 volume PBS prior to the addition of virus (RF or BaL, 10^3 ^tissue culture infectious doses [TCID_50_] as determined in PM-1 cells). Plates were centrifuged for a minimum of 1 hour (room temperature) at 3200 rpm. Unbound virus was washed away with 2 volumes of PBS. Direct virucidal activity was determined by compound pre-treatment of immobilised virus for 1 hour before culture with target cells (PM-1 cells, 4 × 10^4 ^cells/well) in the absence of compound (compound was removed with 4 PBS washes). Receptor mediated blockade activity was determined by the pre-treatment of target cells (1 hour) prior to exposure to immobilised virus in the absence of compound (where compound was removed from treated cells by 4 PBS washes). Attachment/fusion inhibition was determined by the pre-treatment of immobilised virus with test compound prior to the addition of target cells in the presence of compound. Plates were cultured for 10 days, in the absence of media (or compound) replenishment, when viral replication was determined by measurement of RT in culture supernatants. The described assay allows topical administration of candidate compounds: previous studies have demonstrated no difference in compound activity against virus that is either in suspension of immobilised onto plastic (data not shown).

### DC-SIGN binding and transfer assay

To determine whether compounds blocked either virus binding and/or transfer via DC-SIGN, CD4^-^-DC-SIGN^+ ^or CD4^-^-DC-SIGN^- ^Raji cells (0.5 × 10^4 ^cells/well) were treated with test compound for 1 hour at 37°C prior to exposure to virus (HIV-1_RF _or HIV-1_BaL_, 10^4 ^TCID_50 _determined in PM-1 cells) for 2 hours at 37°C in the presence of compound. Compound and unbound virus were removed by washing (4 volumes PBS) and cells either: i) lysed in 1% Triton X-100 to determine the level of virus bound to the cell surface (p24 ELISA); or ii) co-cultured with permissive T cells (PM-1 cells, 4 × 10^4 ^cells/well) to evaluate *trans *infection. Co-cultures were assessed for viral replication by measurement of reverse transcriptase activity following 7 days in culture.

### Culture and HIV infection of human genital tract tissue explants

Cervical explant culture was performed as previously described [12,21,22]. Cervical tissue was obtained from women undergoing planned therapeutic hysterectomy (with written consent as per approval from the local Research Ethics Committee). Cervical tissue comprising both epithelium and stromal tissue was cut into 3 mm explants prior to culture submerged in RPMI 10%. Briefly, explants were pre-treated for 1 hour with test compound prior to exposure to HIV-1_BaL _(10^3 ^– 10^5 ^TCID_50 _determined in PHA-activated PBMC) for 2 hours at 37°C. After incubation with infectious virus and compound, explants were washed with 4 volumes of PBS. Explants were then cultured overnight prior to transfer to fresh plates and further culture for 12–14 days, with 50% media feeds every 2–3 days. Migratory cells present in the overnight culture plate were washed with 2 volumes of PBS and then co-cultured with 4 × 10^4 ^PM-1 cells/well to assess blockade of virus transfer by migrating cells. At the end of the assay, HIV-1 infection was determined by the measurement of p24 in culture supernatants (ELISA): supernatants from explant cultures were assessed using Beckman Coulter p24 ELISA (lower detection limit of 15 pg/ml); supernatants from migratory cell co-cultures were analysed with the less sensitive p24 ELISA from NCI (lower detection limit 300 pg/ml).

### Determination of compound toxicity

Viability of cells and tissue was determined following compound treatment by the principle of MTT (3 [4,5-dimethylthiazol-2-yl]-2,5 dipbenyltetrazolium bromide or thiazolyl blue) dye reduction.

#### i) Cellular toxicity

Following compound treatment (or exposure to HSV-2, see below), ME180 cells were washed and exposed to 0.5 mg/ml MTT in complete DMEM for 2–3 hours. Cells were then solubilised in 98% isopropanol with 2% 2N HCl, and the absorbance at 570 nm determined.

#### ii) Tissue toxicity

Following compound treatment, cervical explants were washed (3 volumes PBS) before submersion in 200 μl MTT (0.5 mg/ml) in complete RPMI for 2–3 hours. Tissue was then blotted to remove excess liquid and tissue weight determined. Explants were transferred into 1 ml methanol and incubated overnight at room temperature in the dark. The absorbance of the MTT-formazan product was determined at 570 nm and the percentage viability per mg tissue calculated by comparing test samples to untreated explants.

### Cytokine detection by multiplex bead immunoassay

Cytokine production was determined by multiplex bead immunoassays (Biosource International Inc., UK) as per manufacturers instructions. Tissue explants were exposed to compound for 2 hours prior to compound removal by washing and overnight culture in the absence of compound. Culture supernatant (50 μl) was assessed for the presence of a panel of 10 cytokines (IL-1β, IL-6, IL-8, TNF-α, GM-CSF, MIP-1α, MIP-1β, RANTES, and MCP-1). Lower limits of detection for each cytokine were generally: IL-1β (7 pg/ml), IL-6 (8 pg/ml), IL-8 (8 pg/ml), TNF-α (6 pg/ml), GM-CSF (16 pg/ml), MIP-1α (15 pg/ml), MIP-1β (19 pg/ml), RANTES (23 pg/ml) and MCP-1 (30 pg/ml). Spiked control samples demonstrated that culture conditions and any residual compound did not interfere with assay sensitivity (data not shown). Plates were read using the Luminex 100 system (Luminex Corp., USA) and data analyzed using Bioplex Manager version 4.0 software (Biorad, UK). Cytokine concentrations present in culture supernatants were determined using non-linear regression analysis.

### HSV-2 infectivity reduction assay

ME180 cells (1.5 × 10^4 ^cells/well) were seeded in 96-well plates and cultured overnight. Cells were exposed to test compound alone (to determine compound toxicity), or virus (approximately 5 × 10^4 ^pfu/well) in the presence of compound (to determine inhibitory effects of the compound) for 1 hour. Compound and unbound virus was removed by washing (3 × 200 μl PBS) and cells cultured in fresh media for 48 hours. Viability was then determined by MTT assay. Whilst a decrease in cell viability in wells exposed to virus reflects viral replication, a reduction in viability following exposure to compound alone indicates toxicity. Viability and infectivity values were calculated as percentage of viability from cells exposed to culture medium alone or percentage of infectivity from cells exposed to virus in the absence of compound.

### Statistical analyses

50% inhibitory concentration analysis was determined using non-linear regression analysis, whilst correlation coefficients were calculated by non-parametric correlation (Spearman) and two-tailed p-value calculation (GraphPad PRISM, GraphPad Software, Inc.). Student's T-tests were performed in Excel (Microsoft Corporation).

## Competing interests

The author(s) declare that they have no competing interests.

## Authors' contributions

PSF participated in the design of the study, carried out anti-viral determinations in cellular and tissue models, determined the pro-inflammatory cytokine response in cervical tissue, completed any statistical analyses and helped draft the manuscript. GSW carried out DC-SIGN based experiments whilst PMMM completed anti-HSV-2 testing of compounds. RJS conceived of the study, participated in its design and coordination and helped to draft the manuscript. All authors read and approved the final manuscript.
